# Association between Malnutrition and Coronary Plaque Characteristics in Patients with Acute Coronary Syndrome: An Optical Coherence Tomography Study

**DOI:** 10.31083/j.rcm2410303

**Published:** 2023-10-23

**Authors:** Haihao Yan, Wenjing Yao, Qian Yang, Congcong Li, Zhenyu Wang, Tianxing Li, Yanhong Li, Kexin Song, Feifei Zhang, Yi Dang

**Affiliations:** ^1^Department of Internal Medicine, Graduate School of Hebei Medical University, 050017 Shijiazhuang, Hebei, China; ^2^Department of Cardiology Center, Hebei General Hospital, 050051 Shijiazhuang, Hebei, China; ^3^Department of Graduate School of Hebei North University, 075000 Zhangjiakou, Hebei, China

**Keywords:** malnutrition, controlled nutritional status score, acute coronary syndrome, plaque characteristic, optical coherence tomography

## Abstract

**Background:**

Malnutrition has a negative impact on patients with arteriosclerotic cardiovascular disease (ASCVD); however, only a few studies have confirmed the effect of malnutrition on atherosclerosis. We aimed to investigate the association between malnutrition and vulnerable plaques via optical coherence tomography (OCT).

**Methods:**

Overall, 142 acute coronary syndrome (ACS) patients were included in this study. Malnutrition was assessed using the Controlled Nutritional Status Score (CONUT), and plaque vulnerability was measured using OCT. Finally, patients were divided into four groups according to their CONUT scores and body mass index (BMI) ≥25.0 or not, to further compare the effects of both factors on plaque characteristics in patients.

**Results:**

OCT results showed that there were significant differences in plaque rupture, thin cap fibroatheroma (TCFA), minimal fiber cap thickness (FCT), thrombus, and macrophage infiltration between different nutritional states [Absent (0–1) vs Mild (2–4) vs Moderate (5–8), plaque rupture: 34.8% vs 52.5% vs 66.7%, *p* = 0.038; TCFA: 10.1% vs 24.6% vs 33.3%, *p* = 0.039; minimal FCT: 125.0 vs 110.4 vs 96.9, *p* = 0.022; thrombus: 50.7% vs 70.5% vs 83.3%, *p* = 0.019]. Multivariate logistic regression showed that malnutrition was a significant predictor of plaque vulnerability. Plaque rupture: CONUT score (odds ratio [OR]: 1.448, 95% confidence interval [CI]: 1.136–1.845, *p* = 0.003), Mild (OR: 1.981, 95% CI: 0.932–4.210, *p* = 0.075), and Moderate (OR: 4.375, 95% CI: 1.048–18.255, *p* = 0.043); TCFA: CONUT score (OR: 1.334, 95% CI: 1.029–1.730, *p* = 0.030), Mild (OR: 3.518, 95% CI: 1.251–9.897, *p* = 0.017), and Moderate (OR: 4.863, 95% CI: 1.019–23.208, *p* = 0.047); and macrophage: CONUT score (OR: 1.343, 95% CI: 1.060–1.700, *p* = 0.015), Mild (OR: 3.016, 95% CI: 1.305–6.974, *p* = 0.010), and Moderate (OR: 4.637, 95% CI: 1.159–18.552, *p* = 0.030). Combined CONUT score and BMI showed an independent association with macrophages in the malnourished and overweight group (OR: 4.010, 95% CI: 1.188–13.537, *p* = 0.025).

**Conclusions:**

Malnutrition is a predictor of vulnerable plaques and is associated with inflammatory progression.

## 1. Introduction

Acute coronary syndrome (ACS) is a common cardiovascular emergency; despite the recent advances in its diagnosis and treatment, cardiovascular disease remains the leading cause of death [1]. Poor dietary quality can adversely affect cardiovascular health [2, 3]. Sze *et al*. [4] and Goldfarb *et al*. [5] have confirmed the association of malnutrition with heart failure and valvular heart disease and have shown malnutrition to be a poor predictor of cardiovascular disease. Previous studies have reported the prevalence of malnutrition in patients with ACS, and patients with different levels of malnutrition showed different mortality rates and differences in the associated risk of future major adverse cardiovascular events (MACE) [6, 7]. Optical coherence tomography (OCT) is now widely used in the management of patients with ACS, and as the highest resolution intracavitary imaging technique available, it allows clear identification of coronary artery structures. Recent clinical studies have shown that OCT can identify high-risk plaque features, including plaque rupture, thin cap fibroatheroma (TCFA), lipid-rich plaques (LRP), and macrophages [8, 9], and that these vulnerable plaques have a significant negative impact on coronary arteries of patients and accelerate the progression of arteriosclerotic cardiovascular disease (ASCVD).

Previous studies have shown that different dietary intakes can influence the coronary plaque vulnerability [10, 11]. However, the intracoronary plaque characteristics in patients who are malnourished have not yet been confirmed by studies; therefore, we aimed to evaluate the plaque characteristics of patients with ACS with different degrees of malnutrition using OCT, to provide more intraluminal imaging evidence for studying the prognostic mechanisms of malnutrition in patients with ACS.

## 2. Method

### 2.1 Study Population

The data of 173 patients with ACS who underwent direct percutaneous coronary intervention (PCI) and OCT at the Department of Cardiology, Hebei General Hospital, between January 2019 and January 2022 were collected for inclusion in this study. Fourteen patients with previous coronary artery bypass grafting, cardiogenic shock, severe hepatic and renal insufficiency, and extremely tortuous coronary artery calcification were excluded. OCT images of 159 enrolled patients with ACS were analyzed, and 17 patients were excluded for the following reasons: poor quality OCT images (n = 4), in-stent restenosis (n = 6), and pre-dilatation before OCT imaging (n = 7). Final OCT images of 142 eligible patients with ACS were analyzed. ACSs, including ST-segment elevation myocardial infarction (STEMI), non-ST-segment elevation myocardial infarction (NSTEMI), and unstable angina, were diagnosed according to current guidelines [12] based on elevated cardiac biomarkers, electrocardiogram (ECG) abnormalities suggestive of myocardial ischemia, clinical signs of angina, or echocardiographic wall motion abnormalities. Patient demographics including sex, age, body mass index (BMI), smoking, laboratory results, and coronary risk factors (hypertension, diabetes mellitus, dyslipidemia, family history of cardiovascular disease (CVD)), were collected.

### 2.2 Nutritional Status

The nutritional status was assessed using the Controlled Nutritional Status Score (CONUT), which has been used since its inception to evaluate the nutritional status of patients with various diseases [13, 14, 15] and combines three indicators of serum albumin, lymphocytes, and cholesterol for systematic scoring, classifying patients as Normal (0–1) or Mild (2–4), Moderate (5–8), or Severe (9–12) malnutrition, with higher scores for a poorer nutritional status.

### 2.3 Coronary Angiography

All participants underwent elective coronary angiography using the standard Judkin technique. Coronary angiography was graded and quantified separately by two independent interventionalists who were unaware of the clinical data.

### 2.4 Optical Coherence Tomography Procedures and Image Analysis

Using the ILUMIEN OPTIS OCT system (Abbott Vascular, Santa Clara, CA, USA), infarct-related arteries were identified by at least two trained cardiologists based on angiographic findings, ECG performance, and appropriate treatment strategies (intracoronary thrombolysis, thrombus aspiration), and the OCT imaging catheter was slowly advanced to the distal end of the culprit lesion, and automatic withdrawal was performed while guiding the catheter for contrast injection. All OCT findings were evaluated by 2 interventionalists who were experienced in film review, and discrepancies in their evaluations were resolved by consultation and analyzed using image analysis software (Abbott, Santa Clara, CA, USA). Patients were graded for infarct-related arteries according to previous studies and consensus criteria [16]; including the plaque type for culprit lesions and plaque components for non-culprit lesions, TCFA, LRP, minimum fiber cap thickness (FCT), maximum lipid arc, reference luminal area (RLA), minimum luminal area (MLA), degree of luminal stenosis, thrombus, microvasculature, calcification, macrophage infiltration, and cholesterol crystals. A non-offending lesion site was defined as a plaque in the infarct-related artery at least 5 mm from the offending lesion. Specific definitions of plaque characteristics are provided in the **Supplementary Material**.

### 2.5 Statistical Analysis

All data were analyzed using IBM SPSS Statistics 26.0 (IBM SPSS Statistics, IBM Corporation, Armonk, NY, USA) statistical software. Categorical variables were expressed as numbers (%) and continuous variables were tested for normality and chi-squared test. Those conforming to normal distribution were expressed as mean ± standard deviation (SD), and non-normal distribution were expressed as median (interquartile range). Patients were classified into four groups according to CONUT scores, as follows: Absent (0–1) or Mild (2–4), Moderate (5–8), and Severe (≥9) groups, and further multiple comparison analysis was performed. Pearson chi-squared test or Fisher exact test were used for categorical variables; one-way analysis of variance (ANOVA) or Kruskal–Wallis test were used for continuous variables; and post hoc test was used when *p* < 0.05. Bonferroni correction was used for multiple comparisons between groups. Multivariate logistic regression was used to analyze predictors of statistically different plaque characteristics (*p* < 0.05). Finally, patients were divided into four groups of nutritionally healthy/normal, nutritionally healthy/overweight, malnutrition/normal, and malnutrition/overweight according to their CONUT scores and BMI ≥25.0 or not, to further compare the effects of both factors on plaque characteristics in patients, with *p* < 0.05 considered a statistically significant difference.

## 3. Results

### 3.1 Baseline Characteristics Based on Malnutrition Grade

Of the 142 patients enrolled, 73 had STEMI (51.4%), 28 had NSTEMI (19.7%), and 41 (28.9%) had unstable angina. Nutritional status was assessed using the CONUT in 69 (48.6%) nutritionally healthy, 61 (43.0%) mildly malnourished, 12 (8.5%) moderately malnourished, and 0 (0%) severely malnourished patients. The total CONUT score ranged from 0 to 8 with a median of 2 (interquartile range: 1–3). Table 1 summarizes the clinical characteristics and imaging results of patients with differing nutritional status. The patients in the Absent group were the youngest (49.0 vs 57.0 vs 59.0, *p* = 0.006), had higher lymphocyte counts (2.22 vs 1.31 vs 0.88, *p* < 0.001), total cholesterol (TC) (4.7 vs 3.5 vs 3.1, *p* < 0.001), triglyceride (TG) (1.7 vs 1.2 vs 1.1, *p* < 0.001), and low-density lipoprotein cholesterol (LDL-C) (3.1 vs 2.2 vs 1.9, *p* < 0.001) levels. The Moderate group had a lower baseline thrombolysis in myocardial infarction (TIMI) flow grade and the serum albumin level was lower (40.7 vs 40.3 vs 34.8, *p* = 0.001) than the other two groups (50.7 vs 45.9 vs 91.7, *p* = 0.014). There were no differences in other clinical parameters. Although diagnoses had no statistical difference (*p* = 0.303), we performed multiple comparisons of CONUT and Bonferroni correction between STEMI, NSTEMI and unstable angina (UA) groups, which found that CONUT was higher in the STEMI group (STEMI 1 vs NSTEMI 2 vs UA 3: 2.32 ± 1.86 vs 1.79 ± 1.52 vs 1.37 ± 1.36, *p* = 0.025. *p*1 vs 2 = 0.814, *p*1 vs 3 = 0.021, *p*2 vs 3 = 0.744).

**Table 1.** **Baseline characteristics of patients with different nutritional status (N = 142)**.

Variables		Absent (n = 69)	Mild (n = 61)	Moderate (n = 12)	*p* value
Male (n, %)		58 (84.1)	56 (91.8)	11 (91.7)	0.366
Age (years)		49.0 (36.5, 58.0)	57.0 (43.0, 66.0)	59.0 (53.0, 64.3)	0.006
BMI (${\mathrm{kg/m}}^{2}$)		25.4 (24.1, 27.4)	25.4 (23.8, 27.4)	22.3 (21.9, 25.6)	0.080
Diagnosis (n, %)					0.303
	STEMI	35 (50.7)	29 (47.5)	9 (75.0)	
	NSTEMI	11 (15.9)	15 (24.6)	2 (16.7)	
	UA	23 (33.3)	17 (27.9)	1 (8.3)	
Hypertension (n, %)		30 (43.5)	34 (55.7)	4 (33.3)	0.216
Current smoking (n, %)		20 (29.0)	16 (26.2)	2 (16.7)	0.668
Dyslipidemia (n, %)		18 (26.1)	11 (18.0)	1 (8.3)	0.280
Family history of CVD (n, %)		10 (14.5)	11 (18.0)	1 (8.3)	0.663
Diabetes mellitus (n, %)		24 (34.8)	23 (37.7)	4 (33.3)	0.924
Initial TIMI flow 0–1 (n, %)					0.014
	0–1	35 (50.7)	28 (45.9)	11 (91.7)	
	2–3	34 (49.3)	33 (54.1)	1 (8.3)	
No. of coronary artery narrowed (n, %)					0.709
	1	36 (52.2)	39 (63.9)	7 (58.3)	
	2	22 (31.9)	14 (23.0)	4 (33.3)	
	3	11 (15.9)	8 (13.1)	1 (8.3)	
Aspirin (n, %)		69 (100)	61 (100)	12 (100)	-
P2Y12 receptor antagonist (n, %)		63 (91.3)	54 (88.5)	12 (100)	0.444
Statins (n, %)		63 (91.3)	56 (91.8)	11 (91.7)	0.995
White blood cells (×${\mathrm{10}}^{9}$/L)		8.71 (6.95, 10.71)	7.2 (5.68, 10.61)	9.44 (6.90, 12.96)	0.108
Neutrophil (×${\mathrm{10}}^{9}$/L)		5.32 (4.11, 7.75)	5.08 (3.75, 8.79)	7.47 (5.39, 11.94)	0.161
Lymphocyte (×${\mathrm{10}}^{9}$/L)		2.22 (1.75, 2.76)	1.31 (1.08, 1.68)	0.88 (0.69, 1.09)	*p* < 0.001
TC (mmol/L)		4.7 (4.2, 5.5)	3.5 (3.0, 4.6)	3.1 (2.4, 4.0)	*p* < 0.001
TG (mmol/L)		1.7 (1.2, 2.9)	1.2 (0.8, 1.9)	1.1 (0.8, 1.2)	*p* < 0.001
HDL-C (mmol/L)		0.97 (0.86, 1.15)	1.01 (0.89, 1.18)	1.01 (0.89, 1.18)	0.237
LDL-C (mmol/L)		3.1 (2.8, 3.7)	2.2 (1.7, 3.0)	1.9 (1.4, 2.6)	*p* < 0.001
Lipoprotein a (mg/L)		171.1 (107.2, 295.2)	193.7 (101.3, 349.3)	183.8 (65.6, 328.3)	0.553
LVEF (n, %)		60.0 (53.0, 63.0)	61.0 (56.0, 66.0)	57.5 (52.0, 61.5)	0.065
Albumin (g/dL)		40.7 (38.9, 42.9)	40.3 (38.0, 43.4)	34.8 (34.2, 39.8)	0.001
Albuminuria (n, %)		12 (17.4)	14 (23.0)	3 (25.0)	0.675

Continuous data are presented as mean ± standard deviation or median (25th, 75th percentile). Categorical data are presented as number (%). BMI, body mass index; STEMI, ST-segment elevation myocardial infarction; NSTEMI, non-ST-segment elevation myocardial infarction; UA, unstable angina; TIMI, thrombolysis in myocardial infarction; CVD, cardiovascular disease; TC, total cholesterol; TG, triglyceride; LDL-C, low-density lipoprotein cholesterol; HDL-C, high-density lipoprotein cholesterol; LVEF, left ventricular ejection fraction; N, total sample size; n, sample size for each group.

### 3.2 OCT Results in Patients with Different Malnutrition Grades

**Fig. 1.**
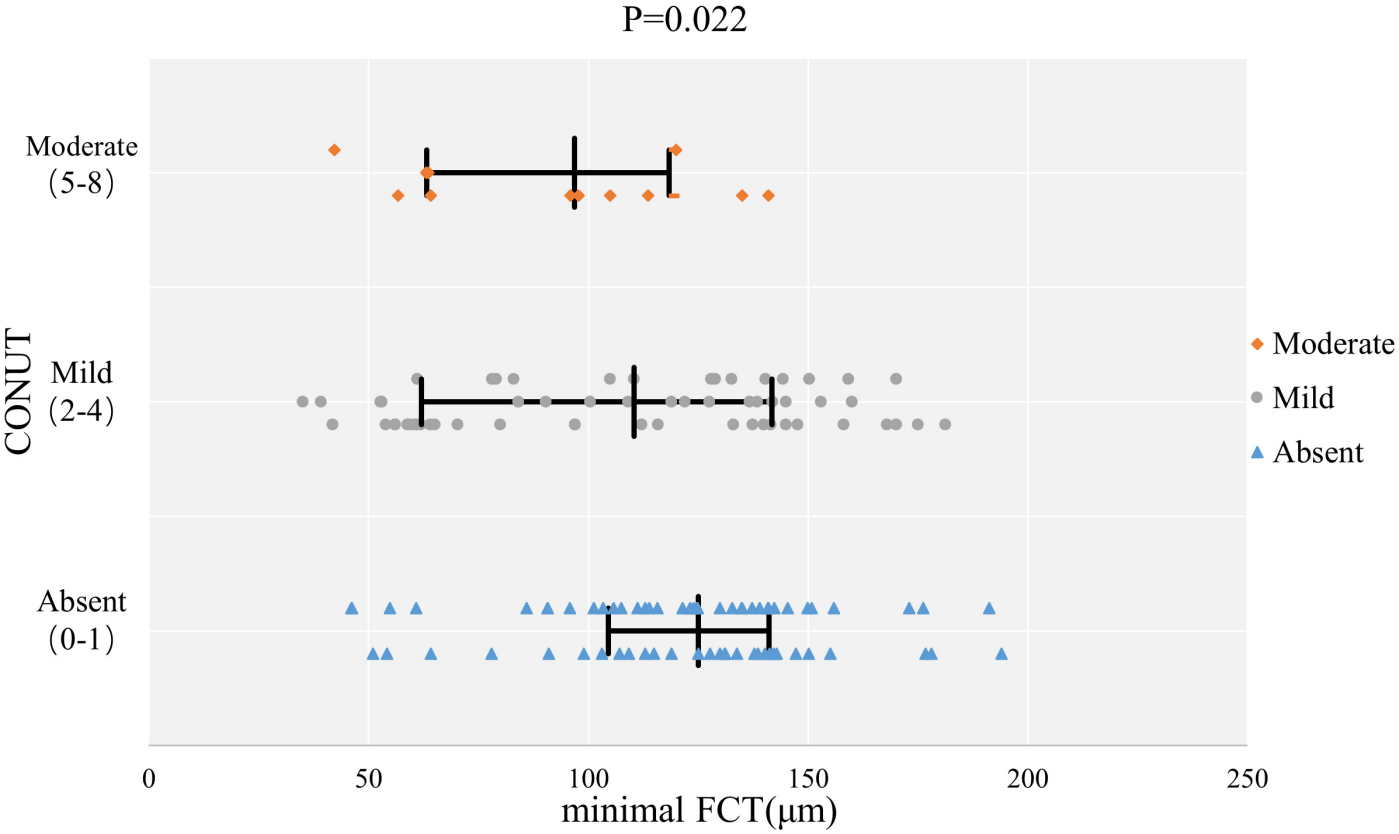
**The comparison of minimal FCT with different malnutrition grades.** Abbreviations: CONUT, Controlled Nutritional Status Score; FCT, fibrous cap thickness.

**Fig. 2.**
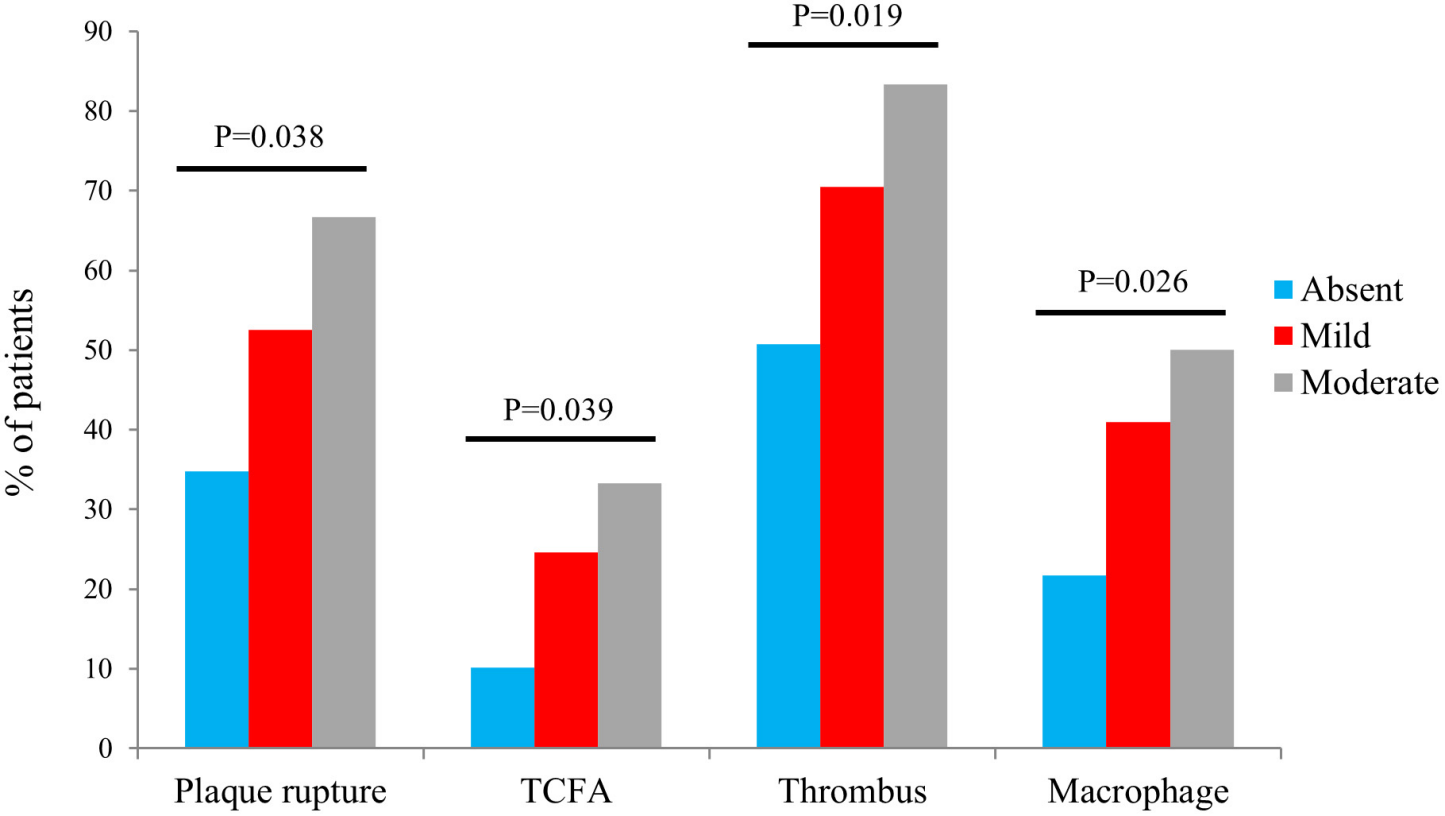
**The comparison of vulnerable plaques with different malnutrition grades**. Abbreviations: TCFA, thin-cap fibroatheroma.

Table 2 shows the comparison of OCT results and indicates that there were significant differences in plaque rupture, TCFA, minimal FCT, thrombus, and macrophage infiltration between the three groups (plaque rupture: 34.8% vs 52.5% vs 66.7%, *p* = 0.038; TCFA: 10.1% vs 24.6% vs 33.3%, *p* = 0.039; minimal FCT: 125.0% vs 110.4% vs 96.9%, *p* = 0.022; thrombus: 50.7% vs 70.5% vs 83.3%, *p* = 0.019). The multiple-group comparison shows that the Absent and Mild groups were statistically significant except for in minimal FCT (*p*1 vs 2 = 0.177), in which no significant difference was observed, and the Mild and Moderate groups had similar plaque characteristics. Figs. 1,2 show the comparison of plaque characteristics, which were statistically different in patients with different malnutrition grades.

**Table 2.** **OCT results in patients with different malnutrition grades (N = 142)**.

Variables	Absent (n = 69)	Mild (n = 61)	Moderate (n = 12)	*p* value	*p*1 vs 2	*p*2 vs 3	*p*1 vs 3
Plaque rupture (n, %)	24 (34.8)	32 (52.5)	8 (66.7)	0.038	0.042	0.366	0.037
LRP (n, %)	39 (56.5)	29 (47.5)	6 (50.0)	0.586	NA	NA	NA
TCFA (n, %)	7 (10.1)	15 (24.6)	4 (33.3)	0.039	0.028	0.528	0.030
Minimal FCT (µm)	125.0 (104.5, 141.1)	110.4 (62.0, 141.7)	96.9 (63.2, 118.4)	0.022	0.177	0.506	0.043
Maximum lipid arc, °	108.1 (68.2, 192.7)	96.0 (76.3, 173.7)	106.7 (79.7, 205.0)	0.931	NA	NA	NA
MLA (${\mathrm{mm}}^{2}$)	2.0 (1.5, 2.7)	1.9 (1.4, 2.5)	2.0 (1.2, 2.4)	0.907	NA	NA	NA
RLA (${\mathrm{mm}}^{2}$)	8.1 (7.2, 9.9)	8.4 (7.2, 9.3)	8.3 (7.8, 9.5)	0.917	NA	NA	NA
Stenosis (%)	76.8 ± 5.1	77.0 ± 5.9	78.8 ± 5.1	0.478	NA	NA	NA
Cholesterol crystals (n, %)	16 (23.2)	11 (18.0)	3 (25.0)	0.728	NA	NA	NA
Thrombus (n, %)	35 (50.7)	43 (70.5)	10 (83.3)	0.019	0.022	0.362	0.036
Microvascular (n, %)	26 (37.7)	24 (39.3)	4 (33.3)	0.923	NA	NA	NA
Macrophage (n, %)	15 (21.7)	25 (41.0)	6 (50.0)	0.026	0.018	0.564	0.039
Calcified plaque (n, %)	28 (40.6)	33 (54.1)	6 (50.0)	0.299	NA	NA	NA

Continuous data are presented as mean ± standard deviation or median (25th, 75th percentile). Categorical data are presented as number (%). OCT, optical coherence tomography; LRP, lipid-rich plaques; TCFA, thin-cap fibroatheroma; FCT, fibrous cap thickness; MLA, minimal lumen area; RLA, reference lumen area; N, total sample size; n, sample size for each group; NA, not applicable.

### 3.3 Predictors of Plaque Vulnerability

We examined the effect of malnutrition on plaque characteristics that were statistically different using a multivariate logistic regression model. The cardiovascular risk factors and medications [17] (age, sex, BMI, hypertension, diabetes mellitus, dyslipidemia, current smoking status, and family history of CVD, statins), CONUT score, and malnutrition grade were included in the multivariable logistic regression analysis when the CONUT score and malnutrition grade were considered as covariate taking separate tests (Table 3). The results showed that malnutrition was a significant predictor of plaque rupture, TCFA, and macrophage infiltration. Plaque rupture: CONUT score (odds ratio [OR]: 1.448, 95% confidence interval [CI]: 1.136–1.845, *p* = 0.003), Mild malnutrition (OR: 1.981, 95% CI: 0.932–4.210, *p* = 0.075), and Moderate malnutrition (OR: 4.375, 95% CI: 1.048–18.255, *p* = 0.043); TCFA: CONUT score (OR: 1.334, 95% CI: 1.029–1.730, *p* = 0.030), Mild malnutrition (OR: 3.518, 95% CI: 1.251–9.897, *p* = 0.017), and Moderate malnutrition (OR: 4.863, 95% CI: 1.019–23.208, *p* = 0.047); and macrophage: CONUT score (OR: 1.343, 95% CI: 1.060–1.700, *p* = 0.015), Mild malnutrition (OR: 3.016, 95% CI: 1.305–6.974, *p* = 0.010), and Moderate malnutrition (OR: 4.637, 95% CI: 1.159–18.552, *p* = 0.030).

**Table 3.** **Multivariate logistic regression analysis for the correlations of malnutrition with plaque vulnerability (N = 142)**.

Variables	Plaque rupture	TCFA	Macrophage	Thrombus
CONUT (as cont.)	1.448 (1.136–1.845)	1.334 (1.029–1.730)	1.343 (1.060–1.700)	1.103 (0.882–1.380)
*p* value	0.003	0.030	0.015	0.390
Malnutrition grade				
Absent	1 [reference]	1 [reference]	1 [reference]	1 [reference]
Mild	1.981 (0.932–4.210)	3.518 (1.251–9.897)	3.016 (1.305–6.974)	1.466 (0.681–3.156)
Mild *p* value	0.075	0.017	0.010	0.329
Moderate	4.375 (1.048–18.255)	4.863 (1.019–23.208)	4.637 (1.159–18.552)	1.380 (0.346–5.507)
Moderate *p* value	0.043	0.047	0.030	0.649

Age, sex, BMI, hypertension, diabetes mellitus, dyslipidemia, current smoking, family history of CVD, statins, CONUT score, and malnutrition grade were included in the multivariable logistic regression analysis. BMI, body mass index; CVD, cardiovascular disease; CONUT, Controlled Nutritional Status Score; TCFA, thin-cap fibroatheroma; N, total sample size.

### 3.4 Effect of Malnutrition Combined with BMI on Plaque Characteristics

We divided the patients into four groups according to the presence of malnutrition (CONUT ≥2) and whether they were overweight (BMI ≥25 ${\mathrm{kg/m}}^{2}$): 17.6% were nutritionally healthy and had normal weight (NN group), 31.0% were nutritionally healthy but overweight (NO group), 24.6% were malnourished but had normal weight (MN group), and 26.8% were malnourished and overweight (MO group). The plaque characteristics of patients with the same nutritional status but different weight classes were compared (Table 4). Among nutritionally healthy participants, patients that were overweight had a higher prevalence of hyperlipidemia (12.0% vs 34.1%, *p* = 0.045), and both had similar plaque characteristics; among malnourished participants, patients that were overweight had a higher prevalence of diabetes (22.9% vs 50.0%, *p*= 0.016), hyperlipidemia (5.7% vs 26.3%, *p* = 0.018), and more macrophages (28.6% vs 55.3%, *p* = 0.021). Multivariate logistic regression analysis of macrophages showed an independent association with macrophages in patients in the MO group compared to patients in the NN group (OR: 4.010, 95% CI: 1.188–13.537, *p* = 0.025), whilst there was no significant difference between patients in the NO and MN groups (NO, OR: 0.666, 95% CI: 0.191–2.328, *p* = 0.525; MN, OR: 1.322, 95% CI: 0.382–4.576, *p* = 0.660) (Table 5).

**Table 4.** **Effect of malnutrition combined with BMI on plaque characteristics**.

Variables	NN (n = 25)	NO (n = 44)	*p* value	MN (n = 35)	MO (n = 38)	*p* value
Male (n, %)	19 (76.0)	39 (88.6)	0.300	30 (85.7)	37 (97.4)	0.166
Age (years)	50.6 ± 19.2	48.1 ± 12.8	0.555	57.6 ± 12.4	53.3 ± 13.3	0.157
Hypertension (n, %)	14 (56.0)	16 (36.4)	0.114	16 (45.7)	22 (57.9)	0.298
Diabetes mellitus (n, %)	9 (36.0)	15 (34.1)	0.873	8 (22.9)	19 (50.0)	0.016
Dyslipidemia (n, %)	3 (12.0)	15 (34.1)	0.045	2 (5.7)	10 (26.3)	0.018
Current smoking (n, %)	6 (24.0)	14 (31.8)	0.491	9 (25.7)	9 (23.7)	0.841
Family history of CVD (n, %)	3 (12.0)	7 (15.9)	0.930	3 (8.6)	9 (23.7)	0.082
Plaque rupture (n, %)	7 (28.0)	17 (38.6)	0.373	18 (51.4)	22 (57.9)	0.579
MLA (${\mathrm{mm}}^{2}$)	2.0 ± 0.7	2.0 ± 0.9	0.857	1.9 ± 1.1	2.1 ± 0.9	0.505
RLA (${\mathrm{mm}}^{2}$)	8.3 ± 1.5	8.2 ± 1.9	0.789	8.2 ± 2.5	8.5 ± 1.9	0.611
Stenosis (%)	76.4 ± 4.5	77.0 ± 5.4	0.688	78.2 ± 6.4	76.5 ± 5.2	0.226
LRP (n, %)	14 (56.0)	25 (56.8)	0.947	17 (48.6)	18 (47.4)	0.918
TCFA (n, %)	3 (12.0)	4 (9.1)	1.000	9 (25.7)	10 (26.3)	0.953
Minimal FCT (µm)	113.6 ± 32.4	127.0 ± 33.0	0.108	104.7 ± 41.4	103.7 ± 40.4	0.920
Maximum lipid arc, °	116.8 (72.5, 184.2)	107.8 (65.9, 205.0)	0.965	109.0 (78.4, 177.5)	96.1 (75.1, 183.0)	0.581
Cholesterol crystals (n, %)	5 (20.0)	11 (25.0)	0.636	7 (20.0)	7 (18.4)	0.864
Thrombus (n, %)	15 (60.0)	20 (45.5)	0.245	26 (74.3)	27 (71.1)	0.757
Microvascular (n, %)	12 (48.0)	14 (31.8)	0.182	13 (37.1)	15 (39.5)	0.838
Macrophage (n, %)	6 (24.0)	9 (20.5)	0.731	10 (28.6)	21 (55.3)	0.021
Calcified plaque (n, %)	10 (32.0)	18 (29.5)	0.941	17 (48.6)	22 (57.9)	0.425

Continuous data are presented as mean ± standard deviation or median (25th, 75th percentile). Categorical data are presented as number (%). BMI, body mass index; CVD, cardiovascular disease; LRP, lipid-rich plaques; TCFA, thin-cap fibroatheroma; FCT, fibrous cap thickness; MLA, minimal lumen area; RLA, reference lumen area; MN, malnourished and normal weight; MO, malnourished and overweight; NN, nutritionally healthy and normal weight; NO, nutritionally healthy and overweight; n, sample size for each group.

**Table 5.** **Multivariate logistic regression analysis of macrophages**.

Variables	Univariate		Multivariate	
	OR (95% CI)	*p* value	OR (95% CI)	*p* value
Age	0.993 (0.969–1.017)	0.562		
Male	0.608 (0.187–1.980)	0.409		
Hypertension	0.675 (0.332–1.373)	0.278		
Diabetes mellitus	1.848 (0.897–3.808)	0.096	1.524 (0.669–3.472)	0.316
Dyslipidemia	1.274 (0.548–2.960)	0.574		
Current smoking	0.675 (0.295–1.543)	0.351		
Family history of CVD	0.969 (0.366–2.570)	0.950		
Statins	0.444 (0.135–1.463)	0.182		
NN	1 [reference]	-	1 [reference]	-
NO	0.814 (0.252–2.635)	0.732	0.666 (0.191–2.328)	0.525
MN	1.267 (0.391–4.101)	0.693	1.322 (0.382–4.576)	0.660
MO	3.912 (1.278–11.973)	0.017	4.010 (1.188–13.537)	0.025

All variables listed were included in the multivariable logistic regression analysis. CI, confidence interval; CVD, cardiovascular disease; OR, odds ratio; MN, malnourished and normal weight; MO, malnourished and overweight; NN, nutritionally healthy and normal weight; NO, nutritionally healthy and overweight.

## 4. Discussion

In this study, for the first time, we observed the morphologic characteristics of lesion types and plaques at non-lesion sites in patients with ACS and different nutritional statuses using OCT. The main findings were as follows: Firstly, malnutrition was present in more than half of the patients with ACS (51.4%) and was associated with incidence of STEMI. Secondly, patients who were malnourished had more vulnerable plaque characteristics than nutritionally healthy patients, and malnutrition, as assessed using CONUT, was a predictor of poor plaque characteristics. Thirdly, both malnutrition and being overweight were not associated with the plaque lipid burden; however, their presence was an independent predictor of macrophages.

Malnutrition is associated with poor prognosis in chronic diseases, such as heart failure, cancer, and kidney disease [4, 18, 19]. The CONUT score, a simple and rapid nutritional assessment tool, has recently been shown to predict adverse events in patients with coronary artery disease (CAD) after PCI based on traditional factors [15, 20, 21]. Malnutrition should be considered in the treatment and perioperative management of patients with cardiovascular disease. Our study showed that more than half of the patients with ACS had malnutrition, and these patients tended to be older in age and have worse perfusion. This could be attributed to the incidence of STEMI. Yokoyama *et al*. [22] demonstrated similar effects of malnutrition on the blood flow of patients with peripheral arterial atherosclerosis, a multivessel disease; the systemic inflammation caused by malnutrition affecting energy expenditure and protein hydrolysis is involved in this process and can lead to cardiac cachexia [4, 17]. However, no patient had severe malnutrition in our study, which we believe is due to the small sample size, strict exclusion criteria, and the very low prevalence of malnutrition, as reported in similar studies [6, 15].

Atherosclerosis is the most critical factor in the development of cardiovascular disease, which usually involves both lipid accumulation and inflammation. Additionally, the instability of the lesioned plaque under certain causative factors may contribute to local thrombosis and consequent vessel occlusion [23]. In our study, patients with a poorer nutritional status were more likely to have plaque rupture and more vulnerable plaques. This finding could explain the potential mechanism by which malnutrition promotes atherosclerosis progression and might also rationalize the occurrence of cardiovascular events regarding intraluminal imaging. Moreover, the impact of TCFA cannot be ignored, as TCFA itself is a prodromal stage of plaque rupture and may be a predictor of the rapid progression of diseased plaques. A prospective, double-blind, international study confirmed that the presence of TCFA in patients with diabetes with normal flow reserve fraction, significantly increased the risk of MACE; and its sub-study showed that the MACE rate was much higher in TCFA than in thick-cap fibrous atheromatous and non-atherosclerotic plaques [24, 25]. Nakagomi *et al*. [26] evaluated the carotid intima-media thickness in patients with chronic heart failure using the CONUT score and confirmed that a poor nutritional status was significantly associated with atherosclerosis and inflammation. Subsequently, Mineoka *et al*. [27] also found that malnutrition contributed to the progression of subclinical atherosclerosis in patients with type 2 diabetes. The pathophysiological mechanisms of malnutrition-induced atherosclerosis are currently recognized as malnutrition-inflammation-atherosclerosis (MIA) syndrome, and the combined involvement of different mechanisms, such as malnutrition-induced inflammatory response and oxidative stress and genetic factors, increases the risk of atherosclerosis [28, 29]. Serum albumin has anti-inflammatory, antioxidant, and antiplatelet effects, and several studies have confirmed that low serum albumin is significantly associated with the development of coronary heart disease and promotes the progression of atherosclerosis, and that this mechanism may be related to the inflammatory response [30, 31, 32]. Cholesterol is a traditional risk factor for atherosclerosis; however, numerous studies have reported the existence of a “cholesterol paradox”, in which low lipid levels may be a marker of advanced disease and systemic inflammatory activation, and contribute to a high vascular inflammatory state [33]. Lymphocytes are essential markers of adaptive immunity, and lower lymphocyte counts are associated with the development of vulnerable plaques [34]. The pro-inflammatory role of malnutrition in the coronary arteries can be further supported by the fact that we observed more macrophage infiltration in the vessels of patients who were malnourished. A study confirmed that the macrophage density on the fibrous cap was negatively correlated with plaque fibrous cap thickness and promoted TCFA formation [35]. LDL particles on the intima require oxidative modification to be taken up by macrophages, which phagocytose these lipoproteins to form foam cells. This further promotes plaque vulnerability as macrophages apoptose and inhibit cytosolic activity, leading to plaque core necrosis as well as poor plaque structural remodeling [23, 36].

BMI also represents the degree of nutritional health of the body and is a risk factor for cardiovascular disease [37]. We combined the CONUT score and BMI and found that malnutrition combined with obesity was an independent predictor of macrophage infiltration, a hyperinflammatory state that appears to indicate an underlying metabolic disease risk. Macrophages may play a vital role in obesity-induced metabolic inflammation, which consequently triggers insulin resistance and metabolic disease [38, 39, 40]. A prospective study showed that metabolically healthy obesity (MHO) is not a steady state and may cause the progression of atherosclerosis with the development of metabolic diseases later in life [41]. Therefore, we believe that early clinical treatment and management of patients who are malnourished and overweight should be conducted to reduce the occurrence of metabolic diseases and thus improve the prognosis. Recent studies have shown that obesity alone is not associated with adverse coronary computed tomography angiography findings and does not increase the risk of MACE in metabolically healthy or unhealthy individuals [42]. Similar results were obtained in our study using OCT analysis of luminal characteristics, with no significant differences in the degree of luminal stenosis and plaque burden in patients with the same nutritional status but differing BMI. The current interpretation is that pericardial and visceral fat volume rather than BMI is associated with atherosclerosis, and two studies by Rodriguez-Granillo *et al*. [43] and Nafakhi *et al*. [44] confirmed that pericardial and visceral fat could assess coronary atherosclerotic burden [45]. Previous studies reported an association between MIA and epicardial adipose tissue (EAT); MIA was associated with an increase in EAT, which explains the involvement of malnutrition in the progression of atherosclerosis, and deserves further investigation [46].

This study has some limitations. First of all, it was a single-center, small study, and the combined OCT examination made the included sample size relatively small. Secondly, before OCT imaging, we had performed some procedure methods (intracoronary thrombolysis, thrombus aspiration) to accurately assess plaque characteristics, which caused underestimation of intracoronary thrombosis. Finally, patients were not followed up to further clarify whether plaque progression and improvement of nutritional status could reduce plaque burden, and there is a lack of imaging evidence for prognostic judgments. Therefore, additional collaborative studies with larger sample sizes and multiple centers are needed to further clarify the impact of malnutrition in atherosclerosis.

## 5. Conclusions

This paper suggests that malnutrition is associated with atherosclerosis and inflammation and is a predictor of vulnerable plaques, explaining the potential mechanisms by which malnutrition affects cardiovascular outcomes. This study provides new insights for the subsequent comprehensive management of patients with CAD.

## Data Availability

The datasets used or analyzed during the current study are available from the corresponding author on reasonable request.

## Abbreviations

ACS, acute coronary syndrome; BMI, body mass index; CI, confidence interval; CONUT, Controlled Nutritional Status Score; HDL-C, high-density lipoprotein cholesterol; LDL-C, low-density lipoprotein cholesterol; MACE, major adverse cardiovascular events; OCT, optical coherence tomography; OR, odds ratio; PCI, percutaneous coronary intervention; TC, total cholesterol; TCFA, thin cap fibroatheroma; TG, triglycerides.

## Supplementary Material

Supplementary material associated with this article can be found, in the online version, at https://doi.org/10.31083/j.rcm2410303.
