# Supplementary material for: Association between Malnutrition and Coronary Plaque Characteristics in Patients with Acute Coronary Syndrome: An Optical Coherence Tomography Study
Source: Rev Cardiovasc Med. 2023 Oct 23;24(10):303. doi: 10.31083/j.rcm2410303 (PMC11273137; doi:10.31083/j.rcm2410303)
Supplement: Supplementary file 1 [file 2153-8174-24-10-303-s1.zip › RCM18566-Supplementary Material-V1.docx]

**Supplementary Appendix:** According to the current consensus criteria for OCT, fibrous plaques have high backscatter and relatively homogeneous signal. Calcified plaques have heterogeneous areas with low signal or well defined borders. Lipid plaques are defined as atherosclerotic plaques with a necrotic core that have low backscatter and high attenuation areas with poorly defined borders and are covered by a fibrous cap. Plaque rupture is defined as disruption of the fibrous cap connecting the coronary lumen to the plaque lumen, exposing the thrombogenic core of the plaque. Plaque erosion is defined as endothelial-only erosion without fibrous cap rupture, with or without thrombus attachment. Thin-cap fibrous atheromatous plaques (TCFA) were defined as plaques with lipid core in ≥1 quadrant and fibrous cap thickness ≤65 μm at the thinnest point. Macrophage accumulation was manifested as signal-rich, distinct or confluent punctate regions that exceeded the intensity of background scattered plaque noise. Microvessels were circular structures with low signal, <250 μm in diameter, identified on more than three consecutive cross-sectional OCT images. Cholesterol crystals were defined as thin linear regions of high intensity, usually associated with fibrous caps or necrotic nuclei. Thrombi are masses located on the luminal surface or floating in the lumen, and OCT defines three types of thrombi: red thrombi, which have high backscatter and high attenuation; white thrombi, which have low and uniform backscatter and low attenuation; and mixed thrombi, which have both characteristics. Minimum fibrous cap thickness was defined as the minimum fibrous cap thickness per image in the presence of lipid plaques. lipid-rich plaques were defined as lipid plaques with a maximum lipid arc >90°. Maximum lipid arc was defined as the maximum lipid arc from the center of the lumen. Minimum lumen area was defined as the minimum lumen area at non-occlusive sites calculated by automated measurement and manual correction. The reference lumen area (RLA) was the average of the maximum lumen area proximal or distal to the lesion, and the percentage of lumen area stenosis was calculated as (1-MLA/RLA)*100%.
